# Co‐Creation and Validation of a Social Media Resource for Mental Health Literacy Among Spanish Adolescents

**DOI:** 10.1111/hex.70469

**Published:** 2025-10-21

**Authors:** Rocío Casañas, Eulàlia Hernández Encuentra, Jenifer Martín, Ferran Lalueza, Mercè Boixadós

**Affiliations:** ^1^ Department of Nursing Universitat de Girona (UdG) Girona Spain; ^2^ Department of Psychology and Education Universitat Oberta de Catalunya (UOC) Barcelona Spain; ^3^ CHM Les Corts Barcelona Spain; ^4^ Universitat Oberta de Catalunya (UOC) Barcelona Spain

**Keywords:** adolescents, co‐creation, digital literacy, digital platforms, help‐seeking, mental health literacy, validation

## Abstract

**Introduction:**

This study aims to design (co‐create) and validate a social media resource for mental health literacy (MHL) based on the interests of adolescents.

**Methods:**

A qualitative descriptive study was conducted between November 2023 and July 2024. The sample comprised 30 third‐ and fourth‐year secondary school students and 10 teachers from three schools in Barcelona, along with 15 health professionals and 7 communication professionals. Co‐creation data were collected through student focus groups, and the resource was validated via an online survey completed by the teachers and health and communication professionals. The findings are reported following the Consolidated Criteria for Reporting Qualitative Research (COREQ) checklist.

**Results:**

As part of the resource co‐creation process, students chose the: (1) theme (non‐suicidal self‐harm, and behavioural problems and anger management issues); (2) social media to be used for dissemination (TikTok's For You Page and/or Instagram); (3) format (1‐ to 2‐min video featuring a young professional providing advice/tips and essential information in a friendly, approachable way); and (4) other aspects (use of trends and subtitles). Validation by professionals confirmed that the content, format and dissemination channel are appropriate for reaching adolescents. They also considered the resource useful, reported that they would use it with adolescents in their care, and said they would recommend it. The overall score for the proposed resource was 4.09 out of 5.

**Conclusions:**

The co‐creation process enabled the design of a social media resource for digital MHL that reflects adolescents' needs and interests. Future research should analyse the potential of such resources to promote MHL among adolescents and define their role within broader interventions.

**Patient or Public Contribution:**

Adolescents, guided by a facilitator, co‐designed a mental health resource that was subsequently validated by professionals in education, communication and health.

## 1 Introduction

According to the World Health Organization (WHO) [1], half of all mental health disorders in adults begin by the age of 18, although most remain undetected and untreated. Emotional problems are common during adolescence, with anxiety and depression being the most frequent, and may present as anxiety attacks, worry, sudden mood swings and even suicide. Suicide is the third leading cause of death among people aged 15–29 [1]. The emotional well‐being and mental health of adolescents is therefore a major concern for national and international organisations, as reflected in reports published in recent decades [1, 2, 3, 4]. WHO [2] recommends psychosocial interventions to improve adolescents' ability to manage their emotions, as well as universal psychosocial interventions that promote good mental health and prevent suicidal behaviour, mental health disorders (such as depression and anxiety), problematic aggressive or oppositional behaviours, and substance abuse.

Mental health is defined as the component of behavioural health that encompasses emotional, psychological and social well‐being [5], as well as the state of well‐being that enables individuals to cope with stressful periods in life, develop their abilities, learn and work effectively, and contribute to their communities [2]. Because this definition includes emotional well‐being, discussions of adolescents' mental health must address this aspect. The European Commission [3] recommends raising awareness and promoting mental health literacy (MHL) through national campaigns and targeted actions. Both WHO and the European Commission stipulate that such programmes should be designed using a holistic approach across various channels and intervention spaces—such as digital media, schools and local communities—and employ diverse strategies for engaging adolescents [3, 6]. Co‐creation is also recommended to optimise the design of digital interventions for this age group [6].

Young people today grow up surrounded by information technologies such as the internet, virtual reality and social media, which they use to seek information, acquire skills and entertain themselves [7]. Digital technologies are also an essential part of their social lives, acting as socialisation agents [8]. Globally, the platforms that adolescents spend the most time on are YouTube, TikTok and Instagram [9], likely because they allow them to communicate with peers, access entertainment in their free time [10], connect with others from anywhere [7], and do so easily via smartphones. At the same time, digital platforms have become a popular channel for promoting MHL among the adolescent population [11, 12, 13].

Kutcher et al. [14] defined MHL as ‘understanding how to obtain and maintain positive mental health; understanding mental disorders and their treatments; decreasing stigma related to mental disorders; and, enhancing help‐seeking efficacy (knowing when and where to seek help and developing competencies designed to improve one's mental health care and self‐management capabilities)’. Jorm [15] offered a narrower definition of MHL as ‘knowledge about mental health problems that underpins action that can benefit mental health’. His formulation explicitly links knowledge to action, specifying that such knowledge must be relevant and support the person's actions and that these actions benefit their mental health. Recent studies indicate that MHL interventions in school settings effectively increase mental health knowledge and improve help‐seeking behaviour among adolescents [16, 17].

Several studies have found that digital animations on social media are among the most effective and cost‐efficient ways to improve MHL in young people [18, 19]. A systematic review by Yeo [20] reported that digital mental health literacy (DMHL) interventions are just as effective as in‐person interventions, achieving optimal mental health outcomes when psychoeducation for DMHL is combined with informal and non‐professional active treatment components, such as skills training and peer support [20].

Recent WHO [6] guidelines stipulate that digital interventions should be co‐created with community stakeholders, including the intended users. The aim is not only to design the most suitable intervention for users' specific needs and preferences [21], but also to tailor it more precisely to meet the intervention's objective, thereby enhancing acceptance and adoption [6]. Co‐creation has already been applied to the design of mobile apps, interventions and information campaigns in the field of child and adolescent mental health [18, 22, 23], as well as to the development of mobile apps and educational interventions for specific health problems [6, 24, 25]. Curran [22], for example, found that a media campaign using innovative messages co‐created by professionals to develop young people's MHL improved their knowledge, attitudes, confidence and willingness to seek help. The study also highlighted the need for continued investment in resources for such campaigns, given their impact on mental health awareness, help‐seeking and stigma reduction among young people.

This study proposes the design of a social media resource for MHL and emotional well‐being, grounded in the interests of Spanish adolescents and validated by professionals in education, health and communication. It forms part of the eHealthLit4Teen project, which examines literacy processes and the dynamics of digital communication around mental and emotional health content on social media used by adolescents in Barcelona. Based on this analysis, we propose a DMHL resource to be implemented through secondary schools.

## 2 Aim

The main objective of this study is to design (co‐create) a social media resource for DMHL based on adolescents' interests and validate it with expert professionals.

## 3 Materials and Methods

### 3.1 Study Design

This qualitative descriptive study used information gathered from the target population. It was conducted in the city of Barcelona, Spain, between November 2023 and July 2024. The Consolidated Criteria for Reporting Qualitative Research (COREQ) [26] were applied (Supporting Information 1).

### 3.2 Sample

Students aged 14–16 in the third and fourth years of compulsory secondary education (*educación secundaria obligatoria*, ESO) at three public and semi‐private schools in Barcelona participated in co‐creating the resource. The same students had previously completed a survey on the use of digital platforms to seek information and training on mental health topics [27].

The resource was validated by three groups of professionals: (1) teachers from the three participating schools; (2) mental health professionals from a child and adolescent mental health centre (CAMHC) in Barcelona, which served more than 1270 young people in 2023; and (3) communication professionals with expertise in social media, including the head of youth communication at Barcelona City Council.

Sampling was done by convenience. Students from each school were selected by their form tutor according to the following inclusion criteria: (1) active use of at least two social media platforms and (2) use of at least one of them for purposes beyond socialising. The same criteria were applied to the teachers. For health professionals, the criteria were: (1) professional work linked to adolescent or youth mental health and (2) more than 5 years' work experience. Communication professionals were included if they had social media management experience.

Based on relevant behavioural change theories, the ideal sample size for information‐gathering studies is 25 participants [28]. This criterion was applied to the student sample. For teachers and health and communication professionals, selection followed homogeneity and heterogeneity criteria aligned with the inclusion criteria. General criteria included age and gender, while participant‐specific criteria were: school type (public or semi‐private) and subject specialisation for teachers; professional role (e.g., nurse) for health professionals; and field of work (e.g., education or consultancy) for communication professionals. Sampling continued until information saturation was reached.

### 3.3 Recruitment and Procedure

Contact was made with the 12 schools where secondary students had previously participated in a survey on the use of digital platforms for information and training on mental health issues. Of these, three schools agreed to participate in this study. The headteacher and the relevant teachers were provided with an information sheet and an informed consent form, along with a separate information sheet for the participating students and their families (mother, father or guardian). The families of all participating students (whether minors or over 16) signed the consent form before the students took part in the study, and the students also signed their own written consent.

The interested teachers signed an informed consent form to participate in a brief, informative online survey. Mental health professionals were recruited via the digital distribution list of the 10 CAMHCs in the city of Barcelona. They were briefed on the study, and those who agreed to take part were sent the survey link and consent form to distribute among their mental health professionals. The first professionals who returned the signed consent form and completed the survey were selected. Communication professionals were recruited through the Professional Association of Journalists of Catalonia (*Col·legi de Periodistes de Catalunya*), which provided a list of collaborators in its training department. These professionals were contacted by email and, following the same procedure as for the mental health professionals, were sent a project briefing, the survey link and an informed consent form. Selection was again based on the order in which professionals provided consent and completed the survey.

All participants were informed that their data would be gathered anonymously, analysed in aggregate, and used solely for academic and research purposes. The project was approved by the Ethics Committee of the Universitat Oberta de Catalunya (reference number 20211209_ehernandez).

The co‐creation process took place over two group sessions held at the schools. The first session identified the themes, content and dissemination format of the resource based on students' interests. The second focused on defining the audiovisual features (logo, colours, music and style) and textual elements (keywords, phrases and hashtags) of the proposed DMHL social media resource. Full scripts for both sessions are provided in Supporting Information S2.

Validation was conducted using an online survey about the resource co‐created with students. Participating professionals were asked to send their replies within 1 week of receiving the survey.

### 3.4 Data Collection

Data on the co‐creation process were collected through focus groups and field notes, while data on the validation process were obtained via an online survey. Two student focus groups were conducted at each school during regular hours, scheduled by the schools at the most suitable day and time for their students and lasting approximately 60–75 min. The sessions were led by three members of the research team (J.M., R.C., and a research assistant [RA]): a PhD‐qualified psychologist and two registered nurses working in community mental health, all women with training and experience in qualitative research. Only the students and researchers were present. The focus groups were audio‐recorded in compliance with ethical requirements, and field notes were taken at the end of each session. Participants had no prior contact with the three researchers and no prior knowledge of them. The objectives of the study and the focus groups were explained at the start of the first session.

The education, health and communication professionals validated the resource through an 11‐question online survey (Table 1) about the resource co‐created with students. Participants viewed the resource at the start of the survey. Seven questions used a five‐point Likert scale (1 = least and 5 = most); two asked respondents to identify the best and worst aspects of the resource from three options (content, format and dissemination proposal); and two were open‐ended (‘What would you add to the resource?’ and ‘Any final comments?’).

**Table 1:** Questions in the online survey used to validate the co‐created resource.

Questions	Score Rate from 1 to 5 (from least to most) the resource
**1**. Is the content suitable for achieving the goal?	1 2 3 4 5
**2**. Is the format suitable for achieving the goal?	1 2 3 4 5
**3**. Is the dissemination channel appropriate for reaching adolescents?	1 2 3 4 5
**4**. Do you think it will be useful?	1 2 3 4 5
**5**. Would you use it with the adolescents you have contact with in your professional environment?	1 2 3 4 5
**6**. Would you recommend it?	1 2 3 4 5
**7**. What overall rating would you give it?	1 2 3 4 5
**Select one of the three response options:**	
**8**. What would you score the highest?	1. Content 2. Format 3. Dissemination proposal
**9**. What would you score the lowest?	1. Content 2. Format 3. Dissemination proposal
**10**. What would you add to the resource?	(Open response)
**11**. Additional comments about the resource	(Open response)

### 3.5 Data Analysis

The student focus groups were transcribed and analysed manually. A paper‐based approach was used due to the manageable volume of data and to ensure the full participation of a visually impaired researcher, who preferred working with printed transcripts. This methodological choice supported inclusive research practices. Coding and categorisation followed an iterative process. Two researchers (R.C. and RA) independently coded the transcripts, identified preliminary themes and then engaged in comparative analysis and discussion to enhance inter‐rater reliability and reach consensus. In cases of disagreement, a third researcher (E.H.) reviewed the transcripts and contributed to the resolution. Researcher triangulation was employed throughout: each transcript was analysed independently, and discrepancies were addressed through repeated analysis and collaborative discussion until consensus was achieved. Finally, transcripts were sent to participants for validation. Once the DMHL resource proposal was developed, it was also sent to all participants (students and professionals) for their feedback.

Information saturation was reached for the data provided by participants [29]. To ensure scientific rigour, the criteria of credibility, reliability and transferability proposed by Graneheim [30] were applied. The COREQ checklist for qualitative research was also used to evaluate the study [26] (see Supporting Information 1). Data from the online survey used in the professional validation process were analysed in SPSS Statistics for Windows, version 29.0 [31], to describe the sample and examine the main variables.

## 4 Results

**Figure 1:**
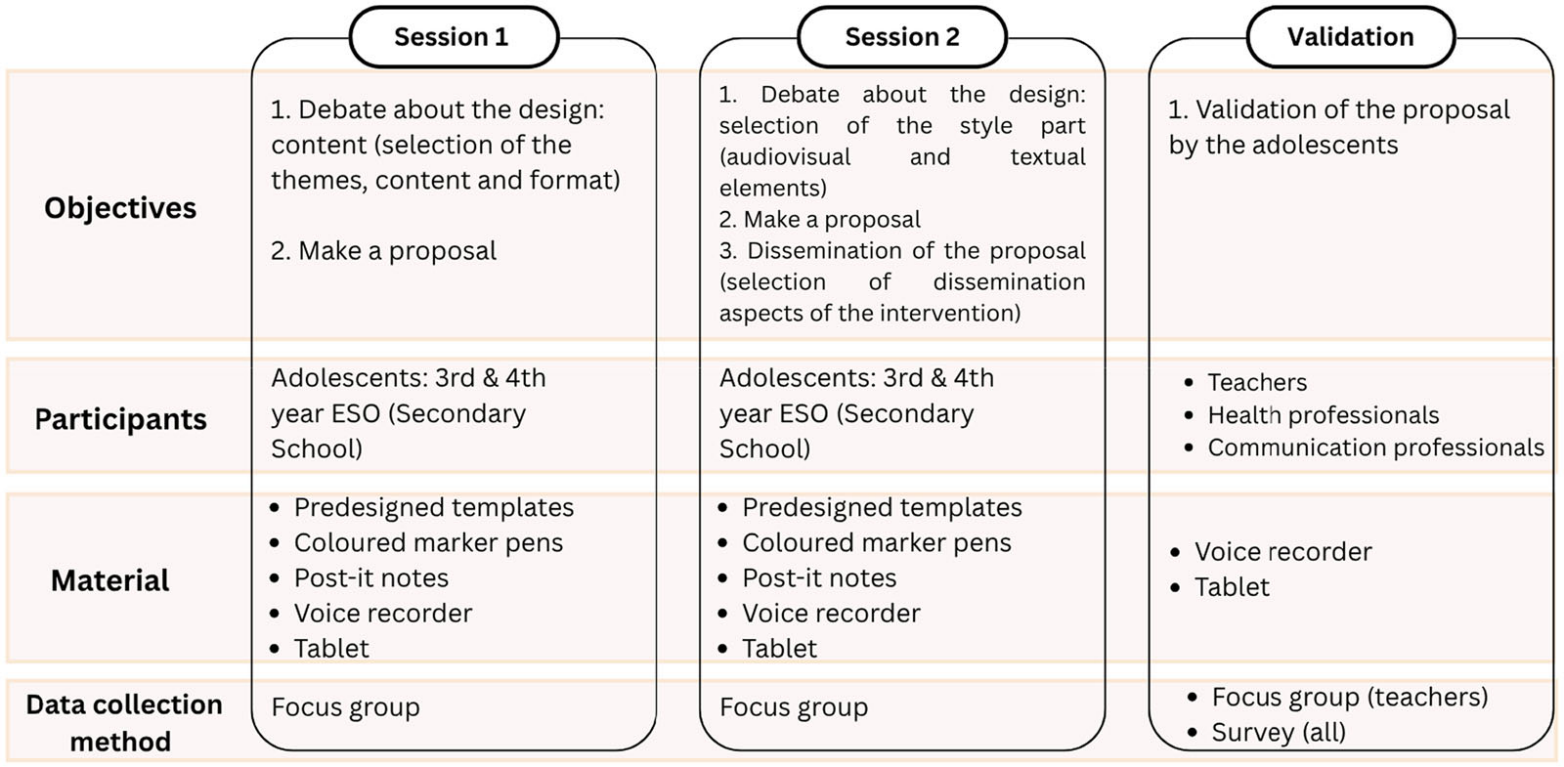
Co‐creation and validation process design.

Figure 1 illustrates the co‐creation and validation processes. In this section, we first present the results of the DMHL resource's co‐creation, followed by its validation by professionals from the fields of education, health and communication.

### 4.1 Resource Co‐Creation Process

Thirty‐six students in their third and fourth years of compulsory secondary education (ESO, in Spain) were invited to participate, with 12 from each of the three selected schools. Of these, 30 students (15 in the third year and 15 in the fourth year) agreed to take part in co‐creating the DMHL resource, comprising 16 girls and 14 boys aged 14–16 (Table 2). All 30 students attended both co‐creation sessions, and none declined to participate.

**Table 2:** Description of the sample in the co‐creation process.

Students (*N* = 30)		
Years	Boys (*n* = 14; 46.67%)	Girls (*n* = 16; 53.3%)
3rd ESO (14–15 years)	5 (16.67%)	9 (30%)
4th ESO (15–16 years)	9 (30%)	7 (23.33%)

Table 3 presents the proposals developed by the student groups during the two co‐creation sessions, organised into seven categories: theme, tone, social media platform, format, type of content, type of publication and other aspects. In ‘other aspects’, students were able to add ideas not included in the proposed categories or in the answer sheet provided to them. Supporting Information 3 contains photographs of the six student‐developed proposals.

**Table 3:** Proposals made by the students during the two co‐creation sessions.

Proposals	School 1 (*n* = 12) 2 groups	School 2 (*n* = 6) 2 groups	School 3 (*n* = 12) 4 groups
Themes	Addictions, substance abuse (2) Behavioural and anger problems (2) Self‐injuring and suicide (1) Anxiety and depression (1)	Addictions, substance abuse (2) Fears (2) Behavioural and anger problems (1) Eating disorders (anorexia and bulimia) (1) Psychotic disorders such as schizophrenia and OCD (1) Available services and programmes (in the community and/or on digital platforms) (1)	Self‐injuring and suicide (4) Eating disorders (anorexia and bulimia) (4) Sexuality (1) Psychotic disorders such as schizophrenia and OCD (3) Child abuse (1) Anxiety and depression (3) Behavioural and anger management problems (3) Loneliness (2) Fears (2) Available services and programmes (in the community and/or on digital platforms) (2) Addictions, substance abuse (2) ADHD/ADD (1)
Tone	Friendly, approachable (2) Serious (1) Other aspects (10): ◦ What are the effects? ◦ How to break addiction ◦ Short‐term advice for breaking addiction ◦ How do I know if I have an addiction? ◦ How to handle bad reactions to drugs ◦ How do you feel when you take drugs? ◦ How to avoid feeling bad ◦ Consequences if you abuse drugs ◦ Easy to understand and clear that the person knows what they're talking about ◦ Reference nurse Jorge Ángel	Humorous (1) Explaining and giving advice (2) Friendly, approachable (2) Focused on risks and what can go wrong (1)	Humorous (2) Explaining and giving advice (2) Friendly, approachable (2) Humorous (but not making it a joke) (1) Acting (1) Focused on risks and what can go wrong (1)
Social networks	TikTok (2) Instagram (1) Other aspects (3): ◦ Instagram Reels (more professional than TikTok) ◦ TikTok: images with instrumental music ◦ Instagram: video with real people	TikTok (2) Instagram (2)	TikTok (4) Twitter (2) Instagram (3) YouTube (1) Ads on social media (1)
Format	Videos (2) Other aspects (4): ◦ Video with photos ◦ Video of the person pointing to different spots where the information/tips appear ◦ Video with images overlaid intermittently (depending on what they are saying) ◦ Description of the structure of the video: 1st image: person with dark circles under their eyes watching someone put ‘sugar’ in their coffee. 2nd image: person removes dark circles and tells the truth: ‘He's not adding “sugar,” it's cocaine’. 3rd image: a link. Phrase: Help yourself see your own reality	Videos (2) Photos (2) Other aspects (3): Brief, specific information	Videos (4) (Short videos, 1 min max.) Photos (2) Memes (1)
Type of content	Practical advice (2) Awareness‐raising messages from influencers (1)	Expert messages (2) Statistics (1) Practical advice (2) Testimony from people affected (1) Other aspects: ◦ What to do if your friend gives you a ‘*whitey’* ◦ 10 things that can happen when you do drugs	Testimony from people affected (4) Practical advice (3) Awareness‐raising messages from influencers (3) Expert messages (2) Collaborations with influencers (1)
Type of publication	Stories (1) Other aspects (8): ◦ Should appear in ‘For You’ on TikTok (and have at least 10–20k likes) ◦ Shared by someone you know ◦ Nurses (individually) ◦ Not organisations (e.g., Gencat, school, council, etc.) ◦ Hours 3–6 PM ◦ The more videos, the better (many publications inspire trust/add validity) ◦ No ads ◦ Hashtags	Publications/Post (1) Stories (2) Other aspects (5): ◦ Hashtags such as: ◦ #fyp ◦ #mentalhealth ◦ #viraltiktok ◦ DO NOT use: #lentejas	Publications/Post (3) Stories (2)
Other aspects	Animations (1) Music (2) Other aspects (3): ◦ Trends ◦ Reggaeton music ◦ Link to the profile where you can ask anonymous questions	Animations (1) Music (2) Interactive (1) Other aspects (2): ◦ Video title: X ◦ More info on (web)	Interactive (2) Animations (3)

As shown in Table 3, students selected the following themes ‘behavioural problems and anger management issues’ (no. of groups = 6); ‘addictions and substance abuse’ (*n* = 6); ‘self‐harm and suicide’ (*n* = 5); and ‘eating disorders (anorexia and bulimia)’ (*n* = 5). They proposed that these should be presented in a ‘friendly, approachable way’ (*n* = 6), by ‘explaining and giving advice’ (*n* = 4). The preferred dissemination channels were ‘Tik Tok’ (*n* = 8) and ‘Instagram’ (*n* = 6). All groups suggested that the resource should be in ‘video format’ (*n* = 8) (1‐ to 2‐min video) or ‘photo format’ (*n* = 4). The proposed content included ‘practical advice’ (*n* = 7), ‘first‐person accounts from those affected’ (*n* = 5) and ‘awareness‐raising messages from influencers’ (*n* = 4). The most frequently chosen publication types were ‘stories’ (*n* = 5) and ‘posts’ (*n* = 4). Students also emphasised the inclusion of ‘animations’ (*n* = 5) and ‘music’ (*n* = 4).

Table 4 presents student responses on the platforms proposed for disseminating the co‐created resource, collected during Session 1 (Activity 1: Debate about the design of the resource) and Session 2 (Activity 2: Dissemination of the proposal). The most representative responses to each question are provided verbatim and translated into English.

**Table 4:** Student proposals for disseminating the co‐created resource.

Questions	Verbatim (translated from Spanish/Catalan) School_x
**1.** Do you remember any videos, posts, stories, reels, posters, news, podcasts, talks and so forth designed to promote mental health and emotional well‐being among young people, or that sought to prevent a particular mental illness? What was it? Where did you find it? What did you do? What do you think of this type of content?	‘*It was a girl talking about her situation while she was doing her make up or something like that … she talks about her personal experience, but doesn't say if she is a psychologist or not … she offers advice’* (S_1) ‘*I came across a guy who talks like nurse Jorge Ángel … he talks in rhymes … but I don't remember his name. It's funny … I don't know if he's a doctor but he talks about health in general. He has quite a lot of followers, more or less like Jorge Ángel. He just came up, I started following him and he started to come up more often’* (S_1) ‘*Famous influencers who post things on TikTok’* (S_3) ‘*TikTok, a TikTok video…’* (S_3)
**2.** What should a publication like this be like? What should it say? Who should deliver this kind of publication? What platform would you use?	‘*There are lots of influencers who make videos about “Types of people when [this happens]” … and then you could say “types of solutions for when [this happens]” … and then you put them in a funny scenario, so people not only enjoy it and find it amusing, but they also share it so others can laugh too’* (S_1) ‘*It should be funny, but not discriminatory humour. There are memes that go viral quickly because they are funny, they are humorous. If you inform people about mental health using humour you might reach more people, because that's how I identify with that*’ (S_1) ‘*I think the most important thing is that they are short videos … they should be short, funny and concise videos’* (S_1) ‘*Video format, short and made by an influencer’ (S*_2) ‘*A short video of maximum 2 min, live, with clear content … and with images, but not drawings’* (S_2) ‘*An entertaining video … that's not too serious, but not like a meme either, it should be a little … for example, a girl doing her make up, the video looks nice … she talks to you in a natural, friendly way’* (S_2) ‘*In an approachable way so that you feel more comfortable and you know that he/she understands you. And explaining and giving advice, because in the end what you are looking for is someone to explain things to you, specific cases, I mean. And advice about what to do if it happens to you’ (S_1)* ‘*It should be interactive like the videos by nurse Jorge Ángel who shows you what to do and doesn't just explain’* (S_1) ‘*Being famous is important for reaching people, and the content must be short … and if you don't use someone famous, they should be backed by someone … the content should be educational, with a message behind it. An influencer who inspires trust, not someone superficial’* (S_2) ‘*A TikTok, a short video of maximum 2 min on YouTube’* (S_2)
**3.** What should it not be like? What would you consider red flags?	‘*Above all, don't make it in two parts, with the second part in another video, because people can't be bothered to watch the second part’* (S_1) ‘*Don't use statistics or deal with the subject too seriously, make it more relaxed and entertaining’* (S_3) ‘*What we don't want is a long video, or that the person doesn't do anything’* (S_2) ‘*The other thing is “Mr Wonderful”‐like phrases, they're awful … a motivational phrase once in a while is fine, but it can't all be based on that kind of thing*…’ (S_3) ‘*If I see a teenager filming themselves with one hand and saying: “5 things I do when I feel anxious,” that is a red flag for me. The simple fact that they don't speak makes me distrust them’* (S_3) ‘*If I see an ad, I skip it. But, sometimes the ads … well now they are showing one that starts like a normal entertainment video but then turns into an ad and you end up watching it’. ‘If there are ads I also skip it’* (S_3)
**4.** Should it use artificial intelligence? How?	‘*If I don't see a real person … maybe if they make me believe it's a real person then yes’* (S_1) ‘*What AI does is answer questions, but it doesn't know how you feel’* (S_1) ‘*But I don't know whether AI is better than being able to ask anonymous questions. AI answers me in a moment but if I ask someone, they may take a week to reply’ (S_2)* ‘*AI gives you clear and quick answers’ (S_2)* ‘*No, because the answers it gives you are automatic, it cannot empathize with you and your problems’ (S_3)* ‘*I think that even though its answers are not personalized, it can give you useful information, and you can decide whether it's helpful or not’ (S_3) ‘It only knows what it has been programmed to know and it can't tailor its answers to your situation’ (S_3) ‘It doesn't understand feelings and it will only give you programmed answers. I don't think that it's very reliable’ (S_3)* ‘*Not for mental health, but for school work, yes’ (S_1)*
**5.** How should we share it so that it reaches teenagers?	‘*The first appearance should be with someone famous … because if it is first launched on a new account, it won't reach anyone, but if you take advantage of someone who has millions of followers it will go much further and later in the post you can tag the hospital and it will have arrived’ (S_2)* ‘*There is a minimum number of likes to make something viral and make it work’ (S_2)* ‘*Normally, people take notice and if you have likes I'll take a look, but if you don't have likes that means that people are not interested and so I skip the video; that's how it usually is’ (S_2)* ‘*Tik Tok, video with music, like Quevedo, and local influencers, that are not as famous, but more real’ (S_3)*
**6.** On which social networks should it be posted?	‘*I think TikTok first, for the algorithm’* (S_2) ‘*I think TikTok is seen by more people’* (S_2) ‘*TikTok and Instagram because they are two social networks that everyone sees, you can look at them at any time of day, whenever, and search for information’ (S_1) ‘because they are the ones we use the most’ (S_1)* ‘*Instagram and TikTok are where most young people usually are, and on YouTube and Twitter too, because normally I think that I spend more time on YouTube reels than TikTok. I prefer YouTube because it's a community that doesn't attack each other as much and is more positive’ (S_2)*
**7.** Who should present the intervention?	‘*I think it's very important that it's a person aged between 20 and 35, because it's like an intermediate age. Not too young for older people and not too old for people of our age … to empathize more, so that the message resonates with you more’ (S_2)* ‘*Any influencer who posts a story saying that they are collaborating with a hospital … that's more believable’ (S_2)* ‘*There are many influencers making videos about “types of people when [this happens]” … and then you could say “types of solutions for when [this happens]” … and then you put them in a funny scenario, so people not only enjoy it and find it amusing, but they also share it so others can laugh too’ (S_2)* ‘*Someone who has experience or knows about the topic. There are lots of influencers posting stories and things about mental health, but they don't really care one way or the other. They aren't trained. And they talk to you about anxiety, depression, etc. In the end, you don't know what to believe’ (S_2)* ‘*It should be something very attractive, not just a person talking to you from behind a desk, but they should be doing something or have something in their hands to give you an example or a metaphor, because it's easier to understand as well as being much more attractive and fun to watch’ (S_1)*
**8.** When should it be posted? At what time of day and on what day? How often should it be posted?	‘*We have obviously chosen the TikTok and Instagram option because they are two social networks that everyone uses, you can look at them at any time of day, whenever, and search for information’ (S_1)* ‘*There is a minimum number of likes to make something viral and make it work’ (S_2)*
**9.** How would you prefer to receive it: directly from the profile that has been created, with ads or without ads, or shared with you by someone else? Should it be disseminated on other media?	‘*If I see an ad, I skip it. But, sometimes the ads … well now they are showing one that starts like a normal entertainment video but then turns into an ad and you end up watching it’. ‘If there are ads I also skip it’ (S_1)* ‘*It should appear in TikTok's “For You” page … with hashtags, for you, lentejas [a viral ironic hashtag meaning not interesting/unwanted content], etc. … but only four, maximum’ (S_1)* ‘*I think that hashtags are only useful for recommending you videos. I would put loads. Well, the top 10 … those that always appear. If it's a video about depression I wouldn't put #lentejas, FYP yes, but not #lentejas’ (S_1)*

Abbreviations: S_1 = school 1; S_2 = school 2; S_3 = school 3.

According to the students' opinions (Table 4), the resource should be a short, concise, educational and humorous video, presented in a friendly and approachable tone. Its aim should be to explain and give advice on ‘what to do if it happens to you’. It should feature an expert (trusted) influencer who can strengthen interaction, attract many followers and likes, and help the video go viral, as user engagement can drive wider dissemination. The students agreed that the video should be posted on TikTok and Instagram (platforms that they use most frequently and can access at any time) and include hashtags so that it reaches a broader audience through algorithmic recommendations.

The students also identified ‘red flags’, or features to avoid. The video should not include statistical data or advertisements, be split into parts, run too long, contain motivational phrases in the ‘Mr Wonderful’ style, or depict an adolescent instructing users on what to do.

Most students felt that artificial intelligence (AI) should not be used to search for information on mental health and emotional well‐being, nor to seek help, as they believed that AI cannot truly understand how adolescents feel. While they acknowledged that they would use AI for an initial information search due to its immediacy (quick and clear responses), they recognised that its answers are automatic, programmed and impersonal, lacking the empathy needed to inspire trust. They reported using AI to help with their schoolwork, but not for mental health matters.

**Figure 2:**
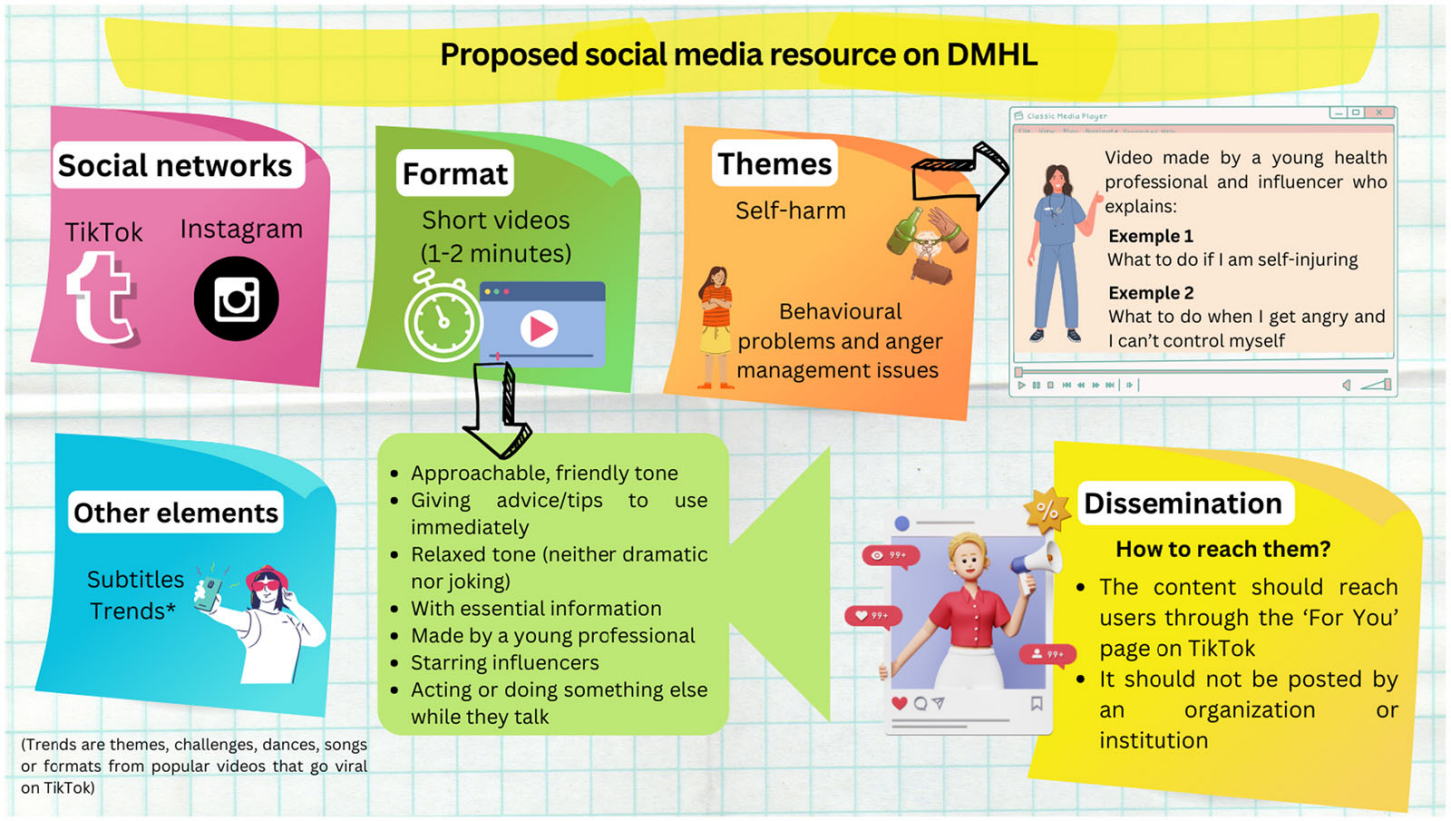
Proposed social media resource on DMHL.

When asked about seeking help for a mental health problem, students said they were unaware of services available in their region. Instead, they indicated that they would first turn to someone they trusted, such as a parent or friend. Figure 2 presents the final proposal for a social media resource on DMHL, co‐created by secondary school students in two group sessions.

### 4.2 Validation Process

Thirty‐two professionals participated in the validation process: 22 women and 10 men, with a mean age of 41.6 (range 29–59). Table 5 presents descriptive information about the sample.

**Table 5:** Description of the sample in the validation process.

Professionals from the education sector		
School. field	Age (years)	Gender
School 1. Language and Literature	52	Women
School 1. Latin and Greek	54	Woman
School 1. Audio‐Visual Culture	36	Men
School 1. Mathematics	42	Man
School 2. Psycho‐Pedagogical Coordinator	39	Men
School 2. Mathematics, Science, Technology	59	Man
School 3. Physics and Chemistry	52	Women
School 3. Spanish Language	40	Woman
School 3. Technology and Economics	49	Woman
School 3. English	52	Woman

Professionals from the health sector (CAMHC)		
Role (*n*)	Age	Gender
Nurse (5)	32	Women
	38	Woman
	29	Woman
	37	Woman
	29	Woman
Clinical psychologist (3)	39	Woman
	30	Men
	40	Man
Psychologist (1)	39	Woman
Psychiatrist (2)	39	Women
Psychiatrist	56	Woman
Social educator (2)	57	Men
	48	Men
Social worker (1)	47	Woman
Occupational therapist (1)	32	Woman

Professionals from the communication sector		
Field	Age	Gender
Local administration	42	Women
Consultancy	43	Woman
Documentation	52	Woman
Education	52	Woman
Education	38	Woman
NGO/Third sector	41	Man

The participants were 10 teachers from three public and semi‐private schools in Barcelona, 15 mental health specialists from a CAMHC in Barcelona, and 7 communication and social media professionals. Their role was to validate the students' co‐created proposal via a single online survey. All responses were complete, and none of the professionals requested that their responses be excluded.

**Figure 3:**
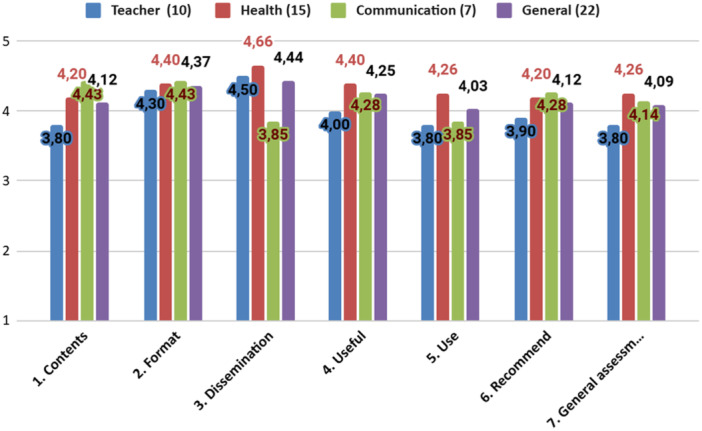
Validation survey results: professional's assessment of the co‐created resource.

Figure 3 shows the responses to the first seven survey questions by professional area (education, health and communication). The professionals judged the content, format and dissemination channels of the resource to be suitable for reaching adolescents, with mean scores of 4.12 (standard deviation [SD] 0.97), 4.37 (SD 0.75) and 4.44 (SD 0.84), respectively. They also considered the resource useful (4.25, SD 0.62), indicated that they would use it with adolescents in their care (4.03, SD 1.06), and said they would recommend it to others (4.12, SD 0.91). The overall assessment of the proposed resource was 4.09 (SD 0.82) out of 5.

All three professional groups rated the format of the co‐created resource similarly (education: 4.30, SD 0.67; health: 4.40, SD 0.82; and communication: 4.43, SD 0.78), in contrast with other aspects. Health professionals rated dissemination highest (4.66, SD 0.62), followed by format (4.40, SD 0.82), usefulness (4.40, SD 0.78), willingness to use it (4.26, SD 0.79) and overall assessment (4.26, 0.59). Communication professionals gave their highest ratings to content (4.43, SD 0.53) and format (4.43, SD 0.78), followed by usefulness (4.28, SD 0.49) and willingness to recommend it (4.28, SD 0.49). Teachers only rated dissemination (4.50, SD 0.97) and format (4.30, SD 0.67) highly.

In response to the question about which aspect of the co‐created resource was strongest, 16 professionals highlighted the chosen format (1‐ to 2‐min video), 10 cited the dissemination proposal (TikTok) and 6 selected the type of content (practical advice). When asked which aspect they considered weakest, 17 professionals pointed to the content (practical advice) and dissemination channel (TikTok), while 9 said the format (1‐ to 2‐min video).

Table 6 details the professionals' responses to the two open‐ended survey questions: ‘What do you think is missing from the proposal?’ and ‘Do you have any further comments to complement your responses?’.

**Table 6:** Responses to the open questions in the online validation survey.

Category	Proposals (by which professional)
**Content**	Add content: resilience, list of mental illnesses (anxiety, difference between anxiety and stress, endogenous disorders, etc.), avoiding social transmission of behaviours (T & HP) Add a health institution that endorses the information (HP) Ask users to put it into practice (HP)
**Format**	Briefer and more concise (CP) Greater depth (T) Use testimonies (CP) Use role models (young, known and independent from organisations) (T) Add tutorials (CP) Face‐to‐face dialogues (T)
**Channel**	Consider YouTube Shorts (CP) Off social media (CP)
**Other**	Regular reminders (HP)

Abbreviations: CP = Communication professional, HP = Health professional, T = Teachers.

## 5 Discussion

The aim of this study was to design (co‐create) a social media resource for mental health and emotional well‐being literacy, based on the interests of secondary school students in Barcelona, through a co‐creation and validation process.

The results indicate that students favour a DMHL resource dealing with specific issues (behavioural problems and anger management issues, addictions and substance abuse, self‐harm and suicide, and eating disorders) in the format of a short (1‐ to 2‐min) video. They preferred content delivered in a friendly, approachable tone by an influencer, performing an action while providing essential information and advice about the topic. The co‐created DMHL resource should also include subtitles, incorporate popular trends and be disseminated on TikTok and Instagram.

During the co‐creation process, students identified important themes for today's adolescents. According to data from 2024, suicide is the third leading cause of death worldwide among people aged 15–29 [32]; in Spain, 174 deaths by suicide were recorded in this age group in 2024 [33]. The prevalence of non‐suicidal self‐harm among adolescents is estimated at 17%, with higher rates among females [34, 35]. These figures underline the urgent need to address adolescent mental health by promoting spaces for dialogue and support aimed at preventing suicide in this population [36].

Behavioural problems and anger management issues also emerged as priorities. Schools have been working on these areas for more than a decade through universal MHL interventions and emotional management programmes [37]. Adolescence is a period of many changes, and the ability to understand and regulate the emotions triggered by everyday situations (some extremely intense, such as rage) is crucial for strengthening emotional well‐being.

Eating disorders, which affect 0.4% of adolescents aged 15–19, are most common among females [1], who often experience greater concerns about their weight and body image [38]. Addictions and substance abuse also pose risks during this stage of life: more than a quarter of adolescents aged 15–19 report drinking alcohol, while cannabis is the most commonly used psychoactive substance among those aged 15–16. These behaviours are often employed as coping mechanisms for emotional difficulties, but can undermine both physical and mental well‐being [1].

On the basis of this evidence, the researchers prioritised two themes—non‐suicidal self‐harm, and behavioural problems and anger management issues—because of their particular significance for adolescents' emotional well‐being and mental health.

One of the most consistent points raised in the co‐creation sessions was the need for the resource (video) to be disseminated by an influencer. Students frequently referred to the Spanish influencer @enfermerojorgeangel (Jorge Ángel Heras), a nurse who communicates about general health topics in an entertaining style and has 6 million followers on TikTok and more than a million on Instagram. As the students explained:

‘It should be interactive like the videos by nurse Jorge Ángel, who shows you what to do and doesn't just explain’./‘Someone who has experience or knows about the topic. There are lots of influencers posting stories and things about mental health, but they don't really care one way or the other. They aren't trained. And they talk to you about anxiety, depression and so on. In the end, you don't know what to believe’.

In recent years, the figure of ‘health influencer’ has become prominent on digital platforms. These are health professionals (e.g., nurses, pharmacists, doctors or psychologists) who combine clinical work with social media communication, often amassing thousands of followers on Instagram and TikTok [39, 40]. They provide practical advice and useful information for young people, using humour and motivational messages [41]. The students said:

‘It should be funny, but not discriminatory humour…. If you inform people about mental health using humour, you might reach more people, because that's how I identify with it’./‘In an approachable way so that you feel more comfortable and know that they understand you. And explaining and giving advice, because in the end, what you're looking for is someone to explain things to you—specific cases, I mean—and advice about what to do if it happens to you’./‘I think the most important thing is that they are short videos … they should be short, funny and concise videos’.

According to students, the resource should be disseminated on TikTok and Instagram so that adolescents can access the information at any time of day:

‘TikTok and Instagram because they are two social media platforms that everyone sees. You can look at them at any time of day, whenever, and search for information … because they're the ones we use the most’./‘I think TikTok first, because of the algorithm’./‘I think TikTok is seen by more people’./‘A TikTok, a short video of maximum 2 min on YouTube’.

Globally, YouTube, TikTok and Instagram are the platforms where adolescents spend the most time [9]. Among Spanish adolescents, TikTok and Instagram are the most popular [8, 39], with an average daily use of 90 min and 70 min, respectively [42].

Similarly, students stressed that the resource should be launched by a well‐known, trusted influencer (such as @enfermerojorgeangel) whose large following and engagement would help it to go viral and reach more young people:

‘…because if it's first launched on a new account, it won't reach anyone, but if you take advantage of someone who has millions of followers it'll go much further, and later in the post you can tag the hospital and it'll have arrived’./‘An influencer who inspires trust, not someone superficial’./‘There is a minimum number of likes to make something viral and make it work’./‘Normally, people take notice—if you have likes, I'll take a look, but if you don't have likes, that means people aren't interested and I skip the video. That's how it usually is’.

Co‐creation is an essential practice for developing digital interventions that are meaningful, relatable and impactful for the target population [18]. Recent studies in the United Kingdom show that co‐created digital animations hold considerable potential for promoting MHL among young people [18, 22]. In our study, co‐creating with adolescents enabled the design of a digital intervention shaped by their concerns and needs, expressed in their own language, featuring their role models, and using the channels and codes that resonate with them.

Overall, the resource designed by the students was rated positively by expert professionals from the education, health and communication sectors. They confirmed that the content, format and dissemination channels were suitable for reaching adolescents and considered the resource useful, reporting that they would use it with the adolescents in their care and recommend it to others.

An analysis of the expert responses shows that teachers rated the resource design less positively than health and communication professionals. This may relate to legislation introduced in Catalonia over the past 2 years restricting mobile phone use in schools and limiting minors' access to social media, to protect them from bullying, inappropriate content and social media addiction. Similar measures have been adopted elsewhere: in Australia and New Zealand, a law now prohibits access to social media for minors under 16, with platforms such as Snapchat, TikTok, Facebook, Instagram and X already identified (although not officially specified) [43]. In Europe, led by France, comparable restrictions are expected for children under 15 who do not have parental permission [44]. Spain's Draft Organic Law for the Protection of Minors in Digital Environments proposes this same ban [45], while Catalonia has issued instructions on mobile phone use in schools, permitting it only during learning activities linked to the teacher's pedagogical objective [46].

At the same time, a counter‐movement led by Australia [47] questions such prohibitions, arguing that they deprive adolescents of opportunities for development and should instead promote meaningful and educational social media use. Today's adolescents, born in the digital era, have grown up surrounded by technology and internet access, making social media central to their communication and socialisation [48]. Overall, the findings of this study highlight the importance of involving professionals who work with children and adolescents in the co‐creation validation process. Their participation not only enhances the quality of interventions but also helps to ensure trust among end users.

In an earlier phase of the research project, young people were asked about their MHL practices. They were not specifically asked about ChatGPT, nor did they mention it [27]. However, data from the Catalan Youth Survey suggest that this technology is beginning to be used by young people [49]. For this reason, during the co‐creation process, students were asked about using AI to learn about mental health topics. They responded that they would not use it to search for information or seek help with a mental health problem. In other words, while students may use AI to help with schoolwork, they do not generally or regularly use it for mental health issues.

Some students acknowledged that they would use AI for an initial information search because of its immediacy (‘It gives you clear and quick answers’./‘If I ask someone, they may take a week to reply’.). At the same time, they were aware that its responses are automatic, programmed and impersonal, meaning that it cannot empathise with how they feel and is therefore untrustworthy:

‘What AI does is answer questions, but it doesn't know how you feel’./‘No, because the answers it gives you are automatic; it can't empathise with you and your problems’./‘It doesn't understand feelings and it will only give you programmed answers. I don't think it's very reliable’.

When seeking help, students said they would turn instead to people they trust, such as parents or friends, speaking to them in person. In the earlier project phase, when asked if they knew about any primary or mental healthcare, social or community resources specifically for young people with mental health problems, they reported being unaware of the resources available in their region [27, 50]. For this reason, even though the students did not request it, the research team decided to include a recommendation to learn about available local resources in the proposed resource.

### 5.1 Strengths and Limitations

The study has two clear strengths. First, the co‐creation process enabled the design of an innovative mental health and emotional well‐being literacy resource for social media that reflected the needs and interests of adolescents. Second, validation of the co‐created resource involved expert professionals from three sectors linked to young people: education, health and communication. As a limitation of the study, although the sample of young people included an equal number of boys and girls and the group sessions allowed for diverse gender‐based contributions, participant gender was not considered in the analysis of contributions to the co‐creation of the final proposal.

### 5.2 Future Research

Future studies should analyse the potential of these resources for fostering MHL among adolescents and define their role within broader interventions. They could also evaluate the specific impact of this resource on young people's emotional well‐being and mental health. This type of social media resource for DMHL can greatly expand access to mental health information, encourage help‐seeking and connect young people with primary and mental health services (e.g., healthcare networks and CAMHCs) previously unknown to them. It is crucial to involve adolescents in promoting mental health and emotional well‐being, and the most effective way is through the social media they engage with every day and that are accessible 24 h a day, 7 days a week.

## 6 Conclusion

Through co‐creation, we designed a resource for TikTok and/or Instagram on mental health and emotional well‐being literacy, taking into account adolescents' interests and preferences. Participating students co‐designed a DMHL resource in short video format (1–2 min), in an entertaining and friendly tone. While presenting essential information on the topic, the influencer engages in some sort of action on screen. Future research should analyse the potential of such resources to promote MHL among adolescents and clarify their role within broader interventions.

## Author Contributions

All authors have contributed to the design and content of the study. Eulàlia Hernández Encuentra and Mercè Boixadós were responsible for the project administration and funding acquisition. Eulàlia Hernández Encuentra and Mercè Boixadós informed the participants about the study and were involved in the recruitment process, introducing the study in the schools. Eulàlia Hernández Encuentra, Mercè Boixadós and Jenifer Martín participated in the data collection. Eulàlia Hernández Encuentra and Rocío Casañas were in charge of the conception and design of the study and major contributors in writing the manuscript, and Mercè Boixadós and Ferran Lalueza were responsible for the analysis and critical revision, providing significant intellectual contributions. All authors read and approved the final manuscript.

## Ethics Statement

The Independent Ethics Committee at the Universitat Oberta de Catalunya approved the project (reference number 20211209_ehernandez).

## Consent

Informed consent was obtained from all the participants in the study. In the case of all students, whether under or over the age of 16, informed consent was obtained from parents or legal guardians; students also provided their informed assent. Written informed consent was requested from the adults and parents or legal guardians of all students participating in the study.

## Conflicts of Interest

The authors declare no conflicts of interest.

## Permission to Reproduce Material From Other Sources

Not applicable.

## Supporting information

3978689 Supplementary materials 1.

3978689 Supplementary materials 2.

3978689 Supplementary materials 3.

## Data Availability

The datasets generated are not publicly available because the study is still ongoing. Further information is available from the corresponding author upon reasonable request.
