# Supplementary material for: Co‐Creation and Validation of a Social Media Resource for Mental Health Literacy Among Spanish Adolescents
Source: Health Expect. 2025 Oct 21;28(5):e70469. doi: 10.1111/hex.70469 (PMC12539285; doi:10.1111/hex.70469)
Supplement: Supplementary file 2 — 3978689 Supplementary materials 2. [file HEX-28-e70469-s003.docx]

**Supplementary material 2: Script for the two group sessions of the co-creation process with students**

| **CO- CREATION PHASE** (participants: 30 third- and fourth-year secondary students) |
| --- |
| **Session 1** |
| **1.** **Presentation and explanation of the aim** of the session (5 min): Co-creation of a DMHL intervention: content of the intervention.  **2. Viewing examples of publications on social networks** (YouTube, TikTok, Instagram) related with emotional wellbeing and mental health, and how to seek information and help for a mental health problem. The viewing is done in groups of four students; each group has an iPad to watch the videos and look at websites (10 min).  **3.** **Activity 1:** **Debate about the design** of the content of the intervention (selection of themes, content and format) (30 min).  We are going to ask you a series of questions about your experience and what you think a publication on mental health should look like, with the aim of having a group debate:  **1-** Do you remember any videos, posts, stories, reels, posters, news, podcasts, talks, etc. designed to promote mental health and emotional wellbeing among young people, or that sought to prevent a particular mental illness? What was it? Where did you find it? What did you do? What do you think of this type of content?  **2-** What should a publication like this be like? What should it say? Who should deliver this kind of publication? What platform would you use?  **3-** What should it not be like? What would you consider red flags?  **4-** Should it use artificial intelligence? How?  **4. Activity 2: Make a proposal** and then share it with the group (20 min).  For this activity you will split into two or three groups and come up with a proposal of what you think would be the best combination of these elements (format, tone, social network, type of content, type of publication, theme and other aspects) for a teenager like you. Please bear in mind that:  - There are seven different types of cards according to the categories (format, tone, social network, type of content, type of publication, theme and other aspects) of the intervention. Each one has a different colour. You should try to explain in detail what each category/element would be like.  - There must be at least one element/colour for each type.  - We also have white cards for each category to note down other ideas you have.  - You can make a sketch of your proposal on the A3 sheet of paper and using the cards.  - You have eight minutes to discuss it with the group and then explain your proposal.  Each team explains their proposal and the members of the other groups can ask questions about the presented proposal.  Once all of the proposals have been presented, you need to choose one together.  **5. Session closing:** 1) Brief summary of the work carried out during the session and final thoughts (10 min). |
|  |
| **Session 2** |
| **1. Explanation of the aim of the session** (5 min): Co-creation of a DMHL intervention: style and dissemination of the intervention.  **2. Activity 1: Debate about the design of the intervention** (selection of the style/appearance of the intervention (audiovisual textual elements) (30 min).  For this activity you will be split into two groups. One group will be in charge of the audiovisual part and the other group will focus on the text.  Audiovisual elements are:   - Logo and colours - Music (or other elements that have come up) - Style of the videos/infographics/profile   Textual elements are:   - Specific words and phrases in the publications - Hashtags   Remember that these should grab the attention of a teenager like you and make them stop and look.  Before splitting into groups, we need to think of a name for the proposal. Do you have any ideas?  You can use paper and markers or create designs using Canva. You have 10 minutes to think about your proposals, then we will share them with each other.  *Audiovisual elements:*  What logo do you suggest?  What colours and style should the publications have?  What music?  What XXX (other elements that have come up)?  *Textual elements:*  What words, phrases and hashtags should be included?  **3. Activity 2: Dissemination of the proposal** (selection of dissemination aspects of the intervention (10 min).  Taking the proposal defined in Activity 1, we now want to publicise it, get people using it and make it go viral! How should we share it so that it reaches teenagers?   - On which social networks should it be posted? - Who should present the intervention? - When should it be posted? At what time of day and on what day? - How often should it be posted? - How would you prefer to receive it: directly from the profile that has been created, with ads or without ads, or shared with you by someone else? - Should it be disseminated on other media?   **4. Session closing:** 1) Brief summary of the intervention proposal designed and final thoughts (10 min) |
