# Supplementary material for: Co‐Creation and Validation of a Social Media Resource for Mental Health Literacy Among Spanish Adolescents
Source: Health Expect. 2025 Oct 21;28(5):e70469. doi: 10.1111/hex.70469 (PMC12539285; doi:10.1111/hex.70469)
Supplement: Supplementary file 3 — 3978689 Supplementary materials 3. [file HEX-28-e70469-s001.docx]

**Supplementary material 3: Image of the proposals created by students in the co-creation phase (Sessions 1 and 2)**

**
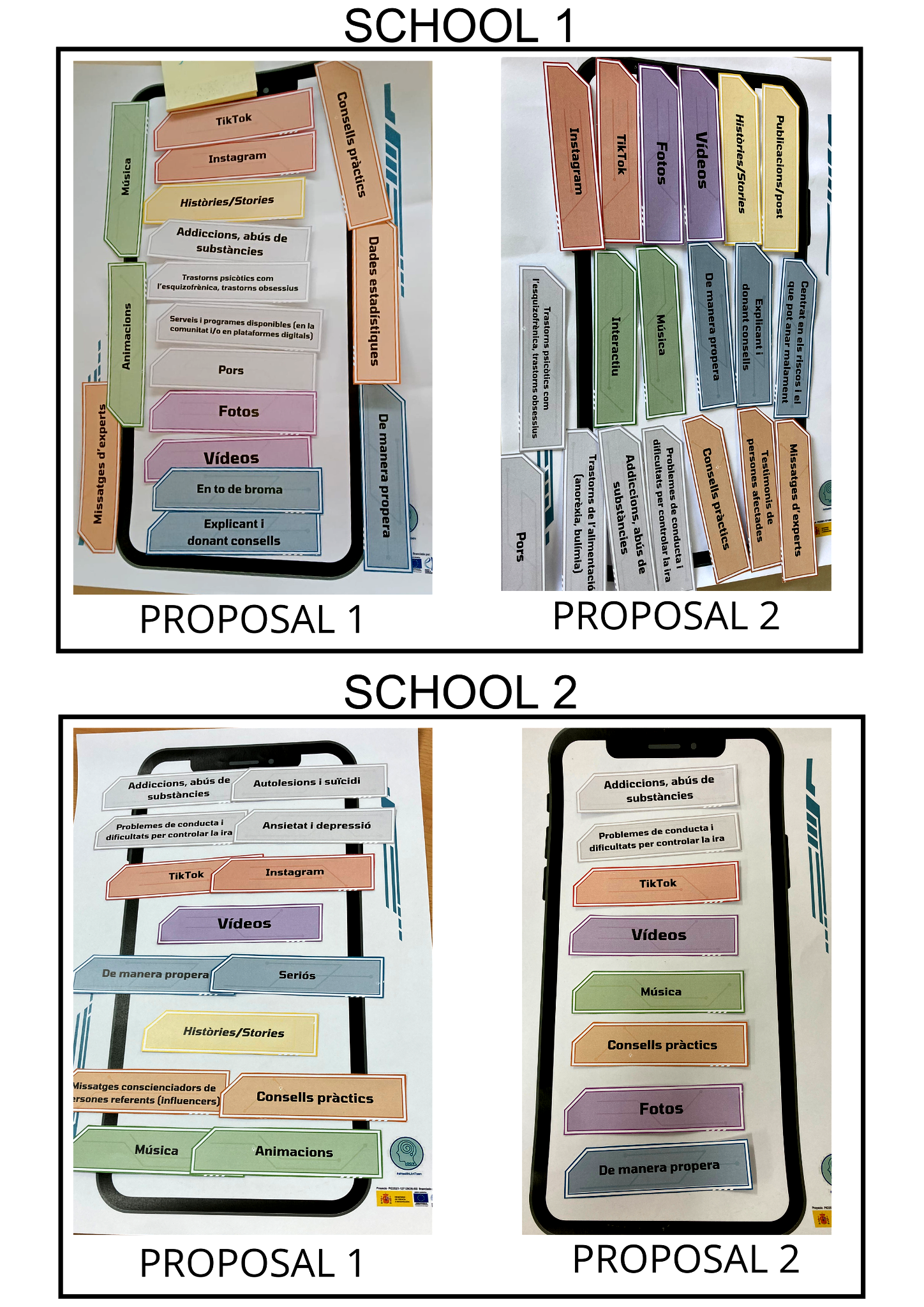
**

**
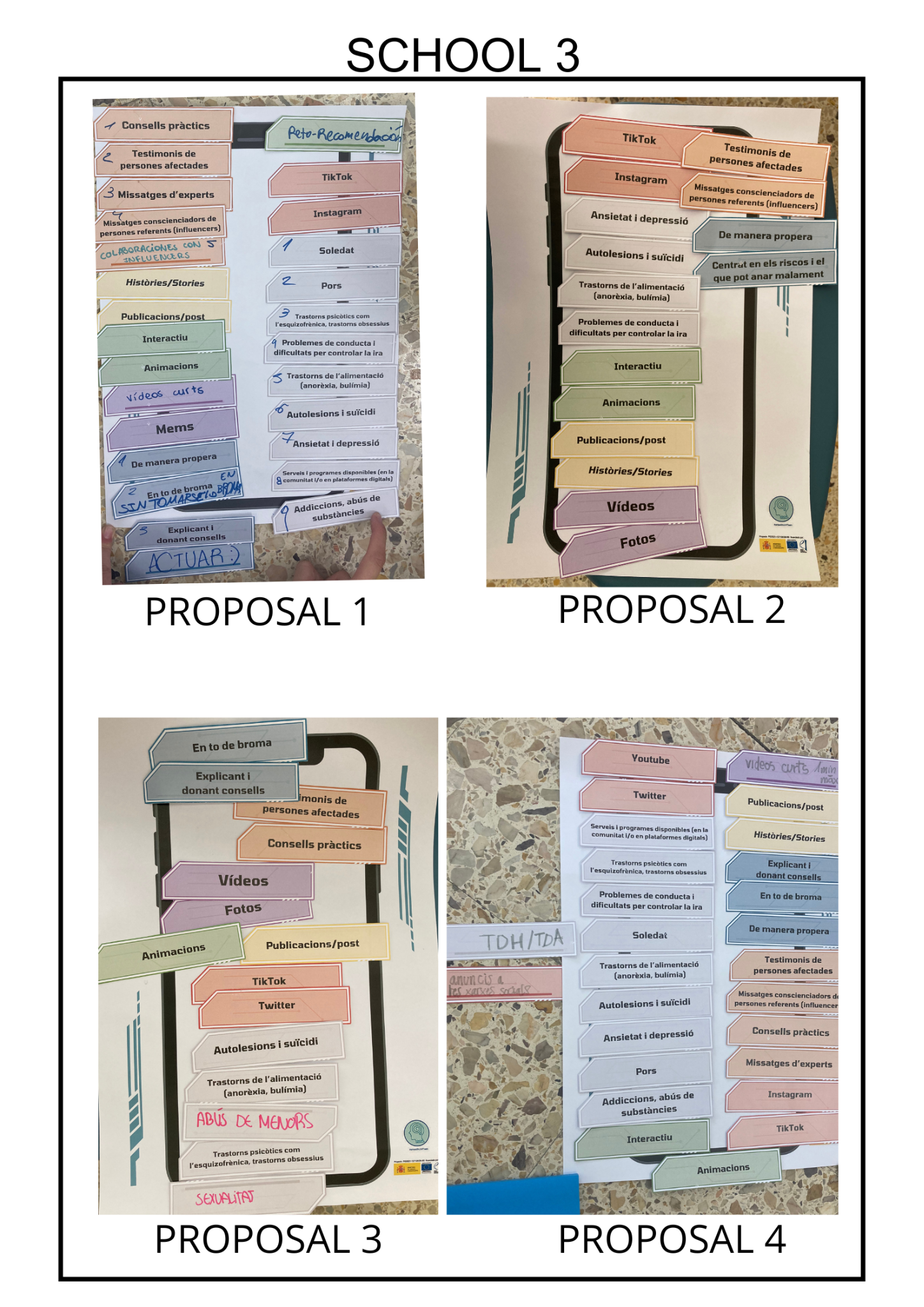
**
